# Live music enhances self-reported audience immersion and physiological synchrony compared to live-streaming

**DOI:** 10.1038/s41598-026-59372-3

**Published:** 2026-06-30

**Authors:** Felix Carter, Guoda Laurinavičiūtė, Emma Harrison, Theodor R. W. Kozlowski, Matteo Antona, Charlotte Butler, Jakub Nawała, Mike Richardson, Danaë Stanton Fraser, Iain D. Gilchrist

**Affiliations:** 1 School of Psychological Science, University of Bristol, 12A Priory Road, Bristol, BS8 1TU UK; 2 School of Computer Science, University of Bristol, Bristol, UK; 3 Department of Psychology, University of Bath, Bath, UK

**Keywords:** Heart-rate synchrony, Live music, Audience experience, Immersion, Livestreaming, Neuroscience, Psychology, Psychology

## Abstract

Before the late 19th century, experiencing music required being physically present with performing musicians. Since then, many methods have attempted to replicate this live experience, most recently livestreaming. It remains unclear to what extent livestreams can replicate the live experience for audience members. To address this, we directly compared audience responses (*N* = 296) to live and livestream performances of two concerts (one jazz, one classical). On arrival at the concert venue, participants were randomly assigned to be physically present in the performance space, or watching a simultaneous, professionally-filmed livestream in a separate room. Questionnaire responses revealed increased reported immersion; increased intent to listen again to the artist and increased intent to return to the venue in the physically present group. This group also had significantly increased levels of heart rate synchrony. In summary, being physically present was associated with an enhanced audience experience compared to viewing a livestream broadcast on all measures. We found no evidence that seating position affects the live experience, whereas for the livestream experience viewing angle (e.g., as altered by camera angle) *does* significantly impact the experience. Future research should examine which perceptual qualities are important for optimising experiences for both live and livestreamed concert audiences.

**Supplementary Information:**

The online version contains supplementary material available at https://doi.org/10.1038/s41598-026-59372-3.

## Introduction

Disruption to the performance arts sector in recent years, in part due to rapid technological advances, the proliferation of access to digital media, and monopolisation of the live music industry^1^, as well as the COVID-19 pandemic (e.g., Whittaker and Wagner^2^ has initiated a shift in how audiences, practitioners and performers engage with livestreaming as a medium for entertainment^3,4^. As a result of this shift, there is increasing interest in how audience experience of viewing livestreamed, or prerecorded concerts, may differ from physical attendance. Aesthetic judgements, emotional response, liking and engagement have been found to be significantly higher for live, compared to prerecorded concerts in a recent study^5^, while others^6^ found positive affect increased significantly more for at-home viewing of a livestreamed performance compared to physical attendance. However, immersion, intellectual stimulation and extramusical social experience were all significantly higher for the live audience. Swarbrick et al.^7^ found musical absorption ratings to be higher for live, compared to livestreamed audiences, but this effect was moderated by fandom (i.e., being a fan) of the artist. Together these findings suggest that there may be a qualitative difference in the way audiences experience these two modes of delivering live music.

Due to the limitations of self-report questionnaire measures (i.e., they are subjective, retrospective and non-continuous), researchers have recently adopted physiological measures to provide more in-depth understanding of audience experiences, including live music. Specifically, interindividual synchrony of physiological responses to stimuli (particularly in heart-rate) appear to be a reliable measure of the strength of affective response to, and cognitive engagement in, numerous forms of audiovisual media. For example, heart-rate synchrony (HRS) has been observed in audiences of theatre (both live^8^; and livestreamed^9^), auditory stories (e.g., Pérez^10^), television^11–13^ and film (e.g., Stuldreher et al.^14^). These studies have tended to report significant relationships between HRS and measures of engagement, whether self-reported narrative engagement and immersion (e.g., Carter et al.^11^, Hammond^12^) or amount of detail of the stimuli subsequently recalled^10,14^. In these studies, HRS is often attributed to the narrative content of the stimuli, with one study demonstrating that even when delivered via distinct perceptual channels (i.e. auditory, visual, or audiovisual) between-group synchrony was not significantly different from within-group synchrony^12^. This suggests that narrative information may be the primary vehicle for audience synchrony. This raises interesting questions around whether music, despite often lacking a clear narrative structure compared to film, television, or theatrical performance, has a similar ability to synchronize sympathetic nervous system activity of audiences.

Multiple studies have now examined physiological synchrony in music concert audiences and their findings suggest the relationship between physiological synchrony and self-reported engagement and immersion may be more ambivalent for live music than for narrative stimuli. Tschacher^15^ found in one study that heart-rate synchrony was positively related to being emotionally moved and inspired by a live classical music performance, while another study Tschacher et al.^16^ found skin conductance and respiration rate synchrony was positively associated with appreciation. However, a subsequent study^17^ found synchrony in heart-rate, heart-rate variability and respiration rate were all *negatively* related to emotional listening (as well as positively related to structural/sound-focused listening, and negatively related to diffuse distraction). A study of recorded music found no relationship between HRS and how moved participants reported feeling^18^.

Musical appreciation has been shown to be reflected in parasympathetic nervous system activity^19^, and live, compared to prerecorded music has been shown to differentially affect activity in multiple neural networks involved in attentional (e.g., frontoparietal regions) and affective (e.g., amygdala) processing^20^. Nonetheless some studies have attributed the finding of synchrony in concert audiences to entrainment to physical features of the music rather than subjective appreciation, engagement or immersion. Hammerschmidt and Wöllner^21^ report that behaviourally synchronizing (i.e. finger-tapping) across different metrical levels alters perception of time, and this effect is moderated by the accuracy of the tapping, suggesting musical features directly modulate attention. Regarding physiological synchronization, Tschacher et al.^16^ found significant synchrony in heart-rate, skin conductance and respiration rate across several classical music pieces, and this synchrony was strongest for the piece with the clearest rhythmic structure (although as a Beethoven composition, it may also have been more familiar to the audience). Czepiel^22^ found synchrony in skin conductance response and respiration rate (but not heart-rate) was related to rhythmic features (i.e., tempo) and increased during salient music events such as changes in loudness, pitch, key, tempo or repetitions of phrases. Similarly, Czepiel et al.^23^ found HRS increased significantly during salient music events (i.e., transition) but interestingly was unrelated to self-reported engagement. While the authors suggest these findings are evidence that physiological synchrony for concert audiences is driven by entrainment, it is interesting to note that audiences who viewed an audiovisual recording of the performance showed significantly higher HRS than those who listened to an audio-only recording. Although it could be argued that the visual information merely strengthened entrainment, this finding does suggest that musical features alone cannot explain physiological synchrony. These mixed results highlight the need for additional research into physiological synchrony among live music audiences, while raising a specific and testable hypothesis regarding its mechanism; if physiological synchrony is driven by entrainment alone, then there should be no difference in synchrony for audiences of live and livestreamed versions of the same concert, as audiovisual content is identical for both.

The current study adopts an experimental design in which ecologically valid audiences for two concerts at an internationally renowned music venue were randomly assigned to view a concert while being physically present in the performance space, or watch a simultaneous, professionally filmed live broadcast (i.e., livestream) in the same venue, but in a separate room, adjacent to the main auditorium. This design ensures that the audience experience of the two groups is as closely matched as possible, right up to the point of condition assignment upon arriving at the venue. This is an important factor as the venue appears to be one of the most important moderators of audience experience of classical concerts^24^.

This study aims to build on previous research by extending the literatures on both the relationship between HRS and self-reported musical engagement/immersion, and the differences in audience experience of live and livestreamed concerts. It also represents the first attempt to address this latter question through use of physiological synchrony measures, which will provide further insight into the mechanisms of physiological synchrony in music audiences. Additionally, by examining audience experience across two concerts of different musical genres (classical and jazz), it extends the literature on physiological synchrony of concert audiences which have previously focused almost exclusively on classical music (e.g., Czepiel et al.^15–17,22^).

## Methods

The data reported here was collected during two concerts held at St George’s Concert Hall, Bristol, UK (www.stgeorgesbristol.co.uk).

### Participants and procedure

Two-hundred and ninety-six participants (170 women, 117 men, 3 non-binary and 6 unreported) participated in the study, which took place at St. George’s Concert Hall, located in the city centre of Bristol (UK). It was built in the 1820s as a Georgian church, and the building was later converted into a dedicated music venue in the late 20th century. It is now widely known for its outstanding acoustics and unique architecture.

In order to obtain an audience that was as reliably representative/ecologically valid of the typical attendees of the venue and of the performances, participants were recruited through St George’s standard ticketing system. The event was advertised and sold to the general public in the usual manner, with information indicating that the performance would be part of a research study on audience engagement. Attendees could choose between a standard ticket, which included participation in the general study, or a discounted ticket that involved additional participation in a heart rate monitoring study. Finally, tickets were sold for two separate performances held on consecutive days. The first event featured a classical trio, while the second featured a jazz trio.

Upon arriving at the venue, participants who had signed-up to wear an electrocardiogram (ECG) heart-rate sensor (Polar H10) were shown how to wear the device and provided a private space in which to fit it. This group of participants were then randomly assigned to either the Auditorium (live) or Studio (livestreamed) condition by drawing a ping pong ball from an opaque bag containing balls of two different colours. The colour of the ball determined whether the participant would be seated in the Auditorium or the Studio. Additionally, to ensure the integrity of the randomisation process, but also to offer a comfortable and enjoyable experience for the audience members, special consideration was given to participants who arrived in groups (family, friends, or other). In such cases, only one draw was performed per group, and all members were assigned to the same venue and seated together during the performance. All participants who had requested not to wear a heart-rate monitor went to the Auditorium. Participants had been made aware that they would be assigned to either the Auditorium or the Studio when they signed up to the heart rate ticket group.

In the Studio, participants watched a professionally recorded livestream of the concert, filmed with 4 BlackMagic Pocket Cinema 4k cameras, and streamed with a Blackmagic system to a Samsung BEA-H Crystal UHD 4K Business 85” Television. Audio for the livestream was captured with Schoeps (https://schoeps.de/en/) cardioid microphones (CCM8 for piano, CCM4 for ambient and cello, CCM22 for additional ambient), Microtech Gefell microphones (https://www.microtechgefell.de/; violin and drums), and additional AKG C414 (akg.com), DPA 4099 (dpamicrophones.com), Schoeps CM4 and Microtech Gefell microphones for drums. Double bass was recorded via direct input from a Gallien Krueger 112 MBX (https://www.gallien-krueger.com) amplifier. The livestream audio was played from a Nova Euphoria System Plus PA system (https://novacoustic.com/). The front row of seats in the Studio was 183.5 cm from the screen. In the Auditorium, the front row of seats was approximately 100 cm from the stage.

The classical concert featured Mithras (mithrastrio.co.uk), a trio based in London, UK, and comprised of piano, cello and violin. The jazz concert featured Yetii (www.alexanderveitch.com/yetii), a trio based in Bristol, UK and comprised of piano, double bass and drums.

Upon its conclusion, participants completed a series of questionnaires (details below). Participants from the Studio were then escorted into the Auditorium for a debrief, followed by an encore. Participants who were in the Studio received a voucher for a future concert of their choice at the venue. Excluding encores (for which heart-rate data were not fully captured), the classical and jazz concerts were 49 min and 49 s, and 58 min and 22 s respectively.

All participants provided informed consent to participate. The study was approved by the School of Psychological Science’s Ethics Committee at the University of Bristol (ethics code 22413) and conducted in accordance with the Declaration of Helsinki. The study was preregistered on the Open Science Framework (OSF; https://osf.io/9cj8e). Additional audience data streams were captured (e.g., thermal imaging and video capture) but these data will be discussed in separate papers.

Informed consent was obtained from all performers (Mithras and Yetii) for the publication of photographs of their performances in this open-access manuscript. The performers were aware that their images would appear in the publication and provided consent for this use.

### Self-report questionnaires

Experience of the concert was measured using several different questionnaires; the Musical Absorption questionnaire^7^, a 4-item Aesthetic Experience questionnaire and 9-item assessment of Musical Immersion used in a comparable previous study by Tschacher et al.^17^, and a single-item measure of immersion^12,13^. We note that while we describe the 9-item questionnaire as a measure of Musical Immersion, the questionnaire is not about acoustic or perceptual musical features per se (although it includes item such as ‘I was immersed in the sound’ and ‘I was captivated by the rhythm’), but also includes items pertaining to higher-level features of the experience, such as appreciation of the musicians’ skill, and emotional and cognitive engagement with the music. This is important to clarify given our position that interindividual synchrony (which we use as a proxy measure of immersion) is not purely driven by entrainment to physical (e.g., acoustic) stimulus properties, but also by factors related to the cognitive and affective response to the experience of the stimulus. Thus, our use of the term Musical Immersion (and our conceptualisation of immersion more broadly) does not pertain to perceptual envelopment alone, as it has been used elsewhere (e.g., Agrawal et al.^25^, but rather as reflective of a psychological state that encompasses a range of cognitive, affective, neural, and physiological processes.

Additionally, musicality was measured with a subset of items from the Goldsmith Musical Sophistication Index (G-MSI^26^. Participants were also asked how familiar they were with the pieces performed; how many times they had previously attended concerts at the same venue; how likely they would choose to pay to see the performers again, and how likely they would choose to listen to pieces performed again. A total of 296 questionnaire responses were collected; 129 for the classical concert (auditorium = 102; studio = 27) and 167 for the jazz concert (auditorium = 141; studio = 26). A full copy of the questionnaire is provided in Appendix A.

### ECG acquisition

Electrocardiogram (ECG) signals were recorded with Polar H10 sensors which features electrodes mounted onto an elasticated strap which is worn around the chest. Data were streamed via Bluetooth to acquisition laptops (Dell Latitude 5440) running Lab Streaming Layer (LSL^27^; and Lab Recorder^28^. To create an LSL inlet for the ECG data streams, modified versions of PolarBand2lsl^29^ were created for each ECG device (with MAC addresses hardcoded). This approach was taken to streamline the process of onboarding participants. Additionally, Bluetooth receivers (TPLink UB500) were connected to each acquisition laptop and placed near participants’ seats to improve stability of the streams. Nonetheless, there was a sizeable loss of ECG data, likely due to a combination of technical issues (specifically with the modified Polarband2lsl code, as well as Bluetooth interference) and potentially incorrect placement of some ECG devices by the participants (although instructions were given, participants fitted devices themselves, and placement was not checked by any researchers as this was deemed to have been too invasive). Audio of the concerts was also captured from each acquisition laptop’s microphone via an Audio Capture app for LSL^30^; this allowed timestamps from each stream to be synchronized to one another as well as the music. As reported in our preregistration, we aimed to record ECG data from 128 participants (32 per condition/concert). However, we did not quite manage to recruit this number (*N* = 111) and data loss due to aforementioned issues resulted in 29 sets of ECG data being lost; 18 in the classical concert (Auditorium *N* = 6; Studio *N* = 12) and 11 in the jazz concert (Auditorium *N* = 6; Studio *N* = 5). Thus, complete ECG data were captured for *N* = 82 participants; 39 participants for the classical concert (Auditorium *N* = 24; Studio *N* = 15) and 43 participants for the jazz concert (Auditorium *N* = 23; Studio *N* = 20).

### ECG preprocessing and analysis

ECG data were recorded at 130 Hz and pre-processed in Matlab (version R2024a). Initially, data were detrended and filtered with both high-pass (1 Hz) and low-pass (30 Hz) third-order Butterworth filters. Due to its efficacy in removing noise from ECG signals while preserving its original properties (e.g^31,32^., a Savitsky-Golay filter was used to identify artefactual noise, with the resulting filtered signal subtracted from the original data. This was a third-order filter with a window size of 71 samples (corresponding to a window of 546ms, chosen on the basis of visual inspection of the filtered signals). To additionally smooth the signal, a moving mean with window size of 500ms was applied.

R-peaks (the sharp upward deflections in ECG that mark ventricular contraction, i.e., the heart “beat”) in the cleaned data were identified with Matlab’s *findpeaks* function, with an adaptive minimum peak height threshold of the median ± 1.5 interquartile range of the signal, and a minimum peak distance of 350ms. Additionally, any peaks whose amplitude was < 50% the height of the previous peak were excluded (as visual inspection revealed that many T-waves were being incorrectly identified as R-peaks). For any recordings where visual inspection revealed the adaptive thresholding was still not fully or exclusively capturing R-peaks, custom peak thresholds were defined. Inter-beat interval (IBI) time series were obtained from these cleaned ECG signals. IBI series were then transformed into heart-rate time-series and linearly interpolated to 1 Hz time-series (i.e., a value in beats per minute for each second of recording) using Matlab’s *interp1* function.

Heart-rate synchrony was analysed through intersubject correlation (ISC) of heart-rate time series using the *SUSY* (Surrogate Synchrony) package in R^33^. This procedure involves performing cross correlations between the heart-rate time-series for each dyad in a dataset (here, dyads were created from pairing each individual with each other individual within the same condition). Data are segmented, and each segment cross-correlated across a number of lags; both segment length and lag are user-defined. We used a segment length of 30 s, and a maximum lag of 5 s. These parameters were chosen for consistency with comparable recent studies (e.g., Tschacher et al.^17^). The correlation coefficients for each segment and lag are Fisher z-transformed and averaged to produce a single mean normalized correlation coefficient per dyad. Our analyses include both the dyad-level averages, as well as individual-level averages.

### Sentiment analysis

Free-text responses to the questionnaire item *“Please use this space to tell us anything else about your experience”* were analysed for sentiment using automated sentiment classification, a machine learning technique that systematically classifies the emotional tone of text. We employed the *nlptown/bert-base-multilingual-uncased-sentiment* model, which is based on BERT (Bidirectional Encoder Representations from Transformers^34^), a pre-trained language model that has learned to understand the meaning and context of words.

We selected this pre-trained model because it has been validated for sentiment classification across diverse text types and requires no custom training on our specific dataset. For each response, the model assigns a sentiment score between 1 (Very Negative) and 5 (Very Positive). For example, the phrase “I felt very well supported” would receive a higher score (closer to 5), whereas “The experience was confusing” would receive a lower score (closer to 1). This automated approach allowed for consistent and objective classification of sentiment across all free-text responses.

### Description of hypotheses and analyses

There were two primary hypotheses being tested in this study. Primarily, we were interested in whether the audience in the livestream (Studio) condition would have a comparable experience (measured via heart-rate synchrony and self-report measures) to the physically present (Auditorium) audience. Additionally, we were interested in the relationship between heart-rate synchrony and self-reported engagement.

## Results

### Main effects of condition and concert on HRS

**Fig. 1:**
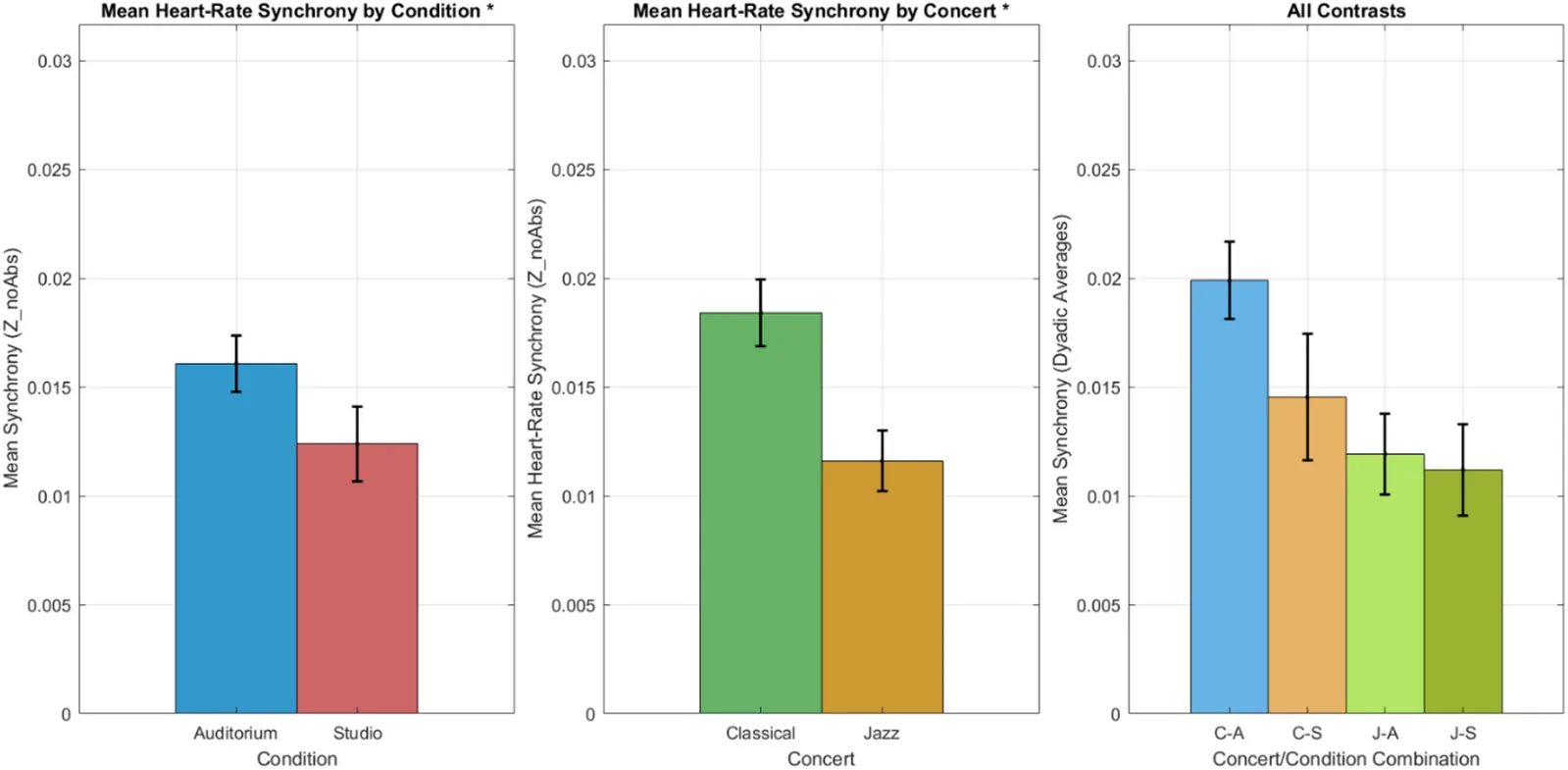
Mean heart-rate synchrony by condition and concert.

Analysis of heart-rate synchrony was performed on dyad-level averages to maximise data retention. Mean heart-rate synchrony by condition and concert are shown in Fig. 1.

Tests for normality indicated significant departures in both independent factors (condition and concert). Kolmogorov-Smirnov tests flagged violations across all levels (all Ps < 0.001), and Anderson-Darling confirmed non-normality for both “Auditorium” (*p* = 0.023) and “Studio” (*p* = 0.001), as well as borderline results for concert level “Yetii” (*p* = 0.074). Given these inconsistencies and observable tail deviations in Q-Q plots, a non-parametric bootstrapped approach (*n* = 10 000) was used to test mean differences in heart-rate synchrony across conditions (Studio/Auditorium) and concerts (Mithras/Yetii) without assuming normality.

In our preregistration we predicted a directional effect of condition on heart-rate synchrony (i.e., that the Studio condition would be associated with significantly lower values than the Auditorium condition). Thus, we conducted one-tailed (bootstrapped) t-tests and report 90% confidence intervals (equivalent to an alpha level of 0.05 for one-tailed t-tests) for the effect of condition, and two-tailed (bootstrapped) t-tests with 95% confidence interval for the effect of concert.

The t-test for condition difference revealed the heart-rate synchrony in the ‘Studio’ condition (M = 0.0124; SD = 0.0294) was significantly lower than in the ‘Auditorium’ condition (M = 0.0161; SD = 0.0297), t(822) = − 1.741, *p* = 0.041, 90% bootstrapped C.I. = [− 0.0072, − 0.0001]. The effect size (Cohen’s d = 0.125) indicated this was a small effect.

The t-test for concert difference revealed the heart-rate synchrony in the ‘Yetii’ concert (M = 0.0116, SD = 0.0292) was significantly lower than in the ‘Mithras’ concert (M = 0.0184, SD = 0.0297), t(822) = − 3.304, *p* = 0.001, 95% bootstrapped C.I. = [− 0.0108, − 0.003]. The effect size (Cohen’s d = 0.231) indicated this was a small-medium effect.

### Condition differences in self-report measures

Condition differences were examined for each experiential self-report measure. Tests of normality (Kolmogorov-Smirnov and Anderson-Darling) and equality of variances (Levene’s test) revealed violation of parametric assumptions for multiple measures. Thus, non-parametric bootstrapping (*N* = 10 000) was used for this analysis. Additionally, Benjamini-Hochberg corrections for multiple comparisons^35^ were applied to control the false-discovery rate (FDR; adjusted p-values are reported here).

**Table 1:** Summary of questionnaire measures by condition and inferential tests of condition differences (Studio vs. Auditorium).

Measure	Studio mean (SE)	Auditorium mean (SE)	t-value	df	*p*-value (adjusted)	95% C.I. (Diff. - bootstrapped)	Cohen’s d
Immersion (single-item)	5.34 (0.21)	5.93 (0.06)	− 2.71	283	0.018	[− 1.0200, − 0.1819].	0.48
Musical Immersion Q (Mean)	5.07 (0.12)	5.41 (0.05)	− 2.70	286	0.011	[− 0.6099, − 0.1018]	0.44
Aesthetic Experience (Emotion)	4.65 (0.2)	5.42 (0.08)	− 3.72	285	< 0.001	[− 1.1836, − 0.3678]	0.60
Aesthetic Experience (Social)	3.61 (0.22)	4.28 (0.09)	− 2.82	284	0.012	[− 1.1359, − 0.2072]	0.45
Absorption (Mean)	4.58 (0.16)	4.93 (0.06)	− 2.08	287	0.049	[− 0.6810, − 0.0194]	0.33
Positively Transformed	4.55 (0.22)	5.11 (0.09)	− 2.39	284	0.025	[− 1.0424, − 0.1138]	0.38

**Fig. 2:**
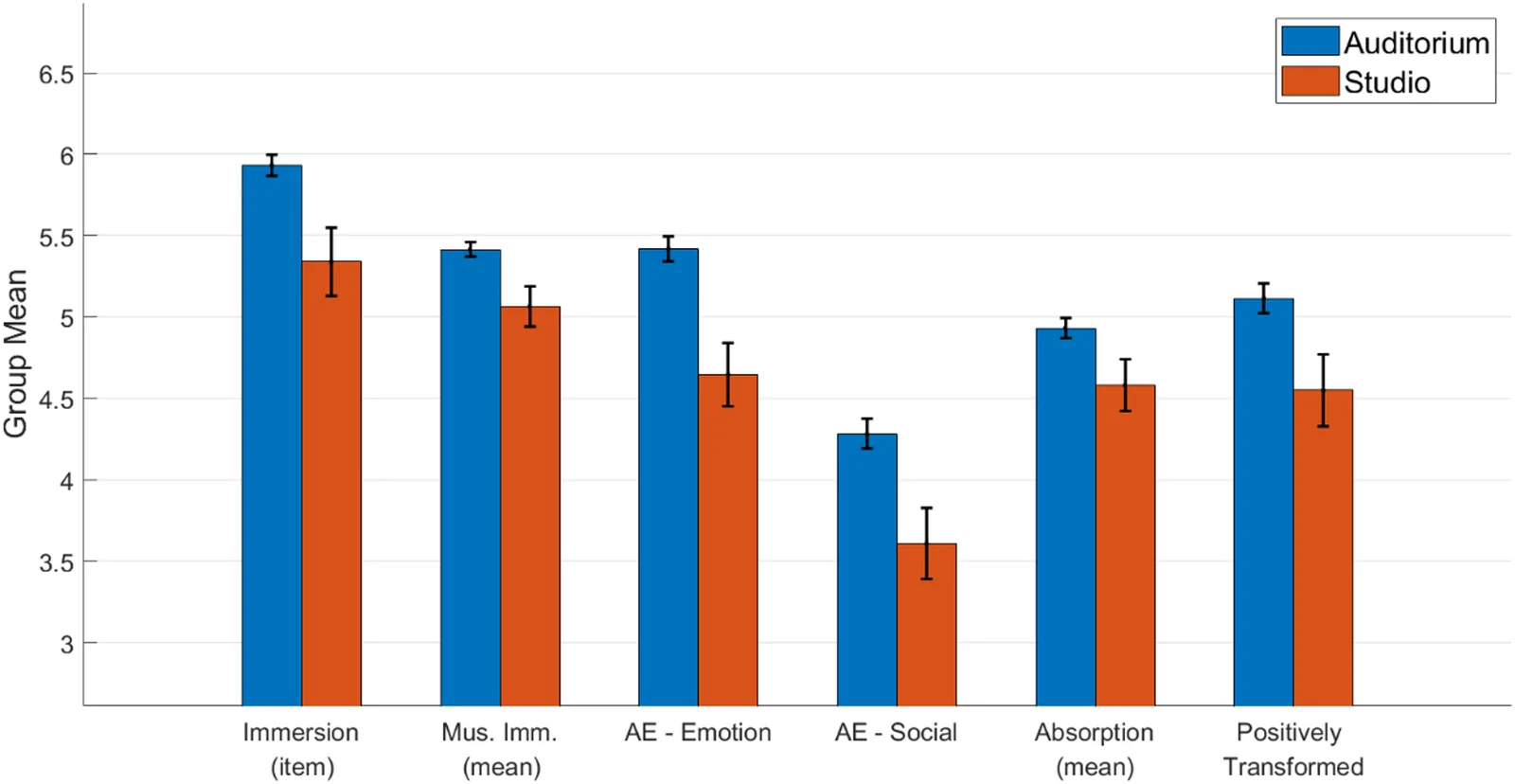
Significant condition differences in questionnaire measures.

**Fig. 3:**
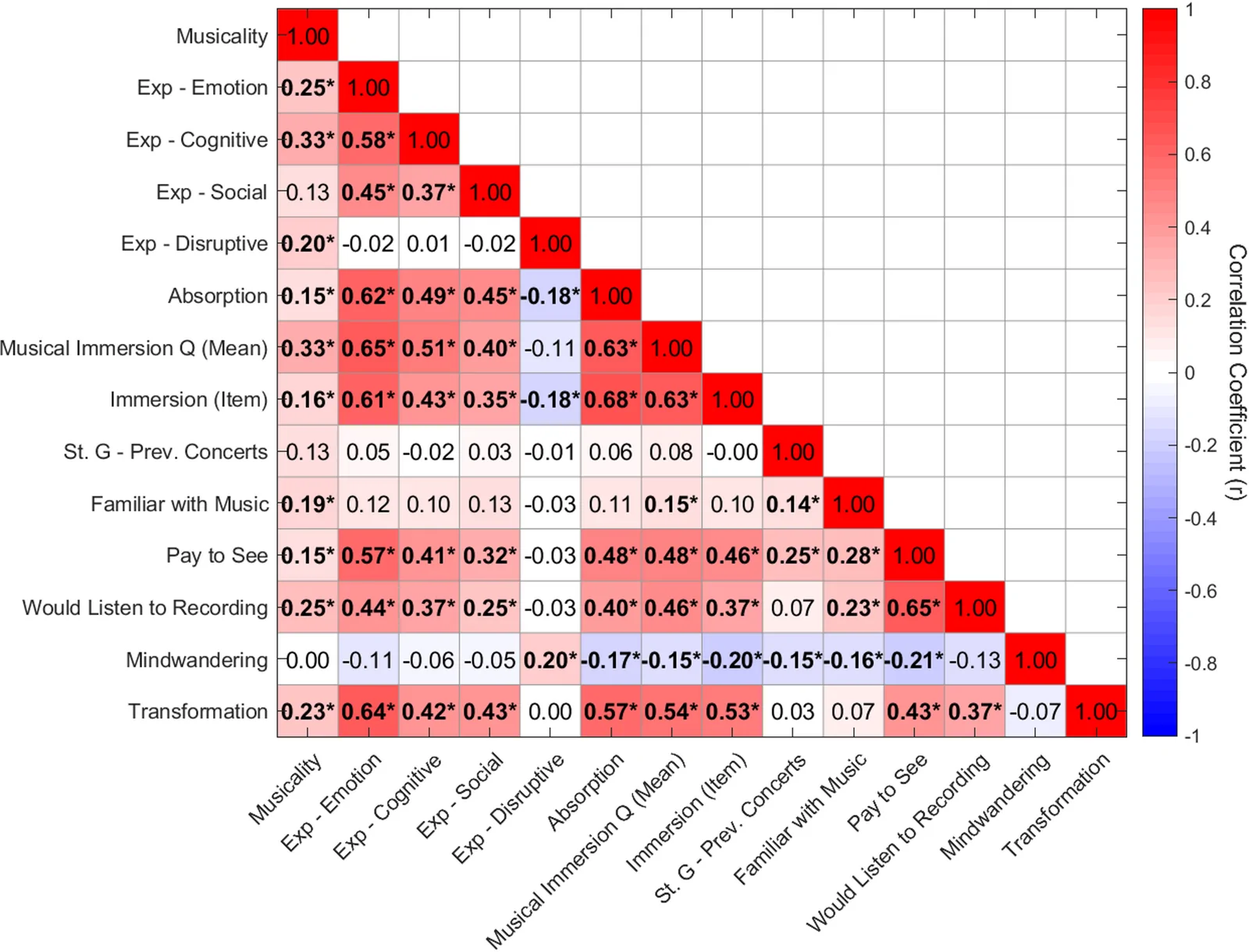
Correlation matrix showing relationships between all questionnaire measures. Values (and colour) indicate the correlation coefficient (Pearson’s R). Significant correlations (i.e. *p* < 0.05; FDR corrected) are indicated by *. Note: Mindwandering is an average of 3 items from the Swarbrick Musical Absorption Inventory (Own World, Daydreaming and Distracted) while Transformation is the vector magnitude of the Positive Transformation and Negative Transformation items (i.e. the square of each item is summed and the square root of the total taken).

For the single-item *immersion* question, values for the Studio condition (M = 5.34, SE = 0.21) were significantly lower than the Auditorium condition (M = 5.93, SE = 0.06); t(283) = − 2.71, *p* = 0.018, 95% bootstrapped C.I. = [− 1.0200, − 0.1819]. Mean scores for the Musical Immersion questionnaire were also significantly lower for the Studio condition (M = 5.07, SE = 0.12) than the Auditorium condition (M = 5.41, SE = 0.05) t(286) = − 2.70, *p* = 0.011, 95% bootstrapped C.I. = [− 0.6099, − 0.1018]. Aesthetic Experience (Emotion) scores were significantly lower in the Studio condition (M = 4.65, SE = 0.20) than the Auditorium condition (M = 5.42, SE = 0.08); t(285) = -3.72, *p* < 0.001, 95% bootstrapped C.I. = [− 1.1836, − 0.3678]. Aesthetic Experience (Social) scores were significantly lower in the Studio condition (M = 3.61, SE = 0.22) than the Auditorium condition (M = 4.28, SE = 0.09); t(284) = − 2.82, *p* = 0.012, 95% bootstrapped C.I. = [− 1.1359, − 0.2072]. Absorption scores were also significantly lower in the Studio condition (M = 4.58, SE = 0.16) than the Auditorium condition (M = 4.93, SE = 0.06); t(287) = − 2.08, *p* = 0.049, 95% bootstrapped C.I. = [− 0.6810, − 0.0194]. Transformation (positive) scores were also significantly lower in the Studio condition (M = 4.55, SE = 0.22) than the Auditorium condition (M = 5.11, SE = 0.09); t(284) = − 2.39, *p* = 0.025, 95% bootstrapped C.I. = [− 1.0424, − 0.1138]. These results are shown in Table 1/Fig. 2. Overall correlations (i.e. collapsed across entire sample) between all questionnaire measures are shown in the correlation matrix below (Fig. 3).

The above analysis was repeated for the data split by *concert* rather than *condition*. No significant differences in any outcome variables survived FDR correction (all adjusted *p* > 0.05).

### Predicting HRS from self-report measures

To examine whether individual mean heart-rate synchrony was related to our self-report measures, we conducted a backwards stepwise regression to predict heart-rate from the self-report measures which were aggregated as described in *Methods.* The list of initial predictors used in this model are given in Appendix B.

For each iteration of the model, the weakest predictor (that with the largest p-value) was removed if its Variance Inflation Factor (VIF) was > 5 (indicating potential collinearity), likelihood ratio p-value of the latter model was above 0.05 (i.e. removing the predictor did not significantly worsen model fit) and if removing the predictor did not increase Akaike Information Criterion (AIC) by 8, or Bayesian Information Criterion (BIC) by 6. These thresholds were chosen as changes above these provide strong evidence against the candidate model (Fabozzi et al., 2014). In other words, by allowing AIC/BIC to increase by up to 6 and 8 respectively for each iteration of the model, we emphasize model simplicity by allowing non-significant predictors to be removed provided they do not significantly decrease model fit. Two participants were not included in the regression due to missing values in one or more variables.

**Fig. 4:**
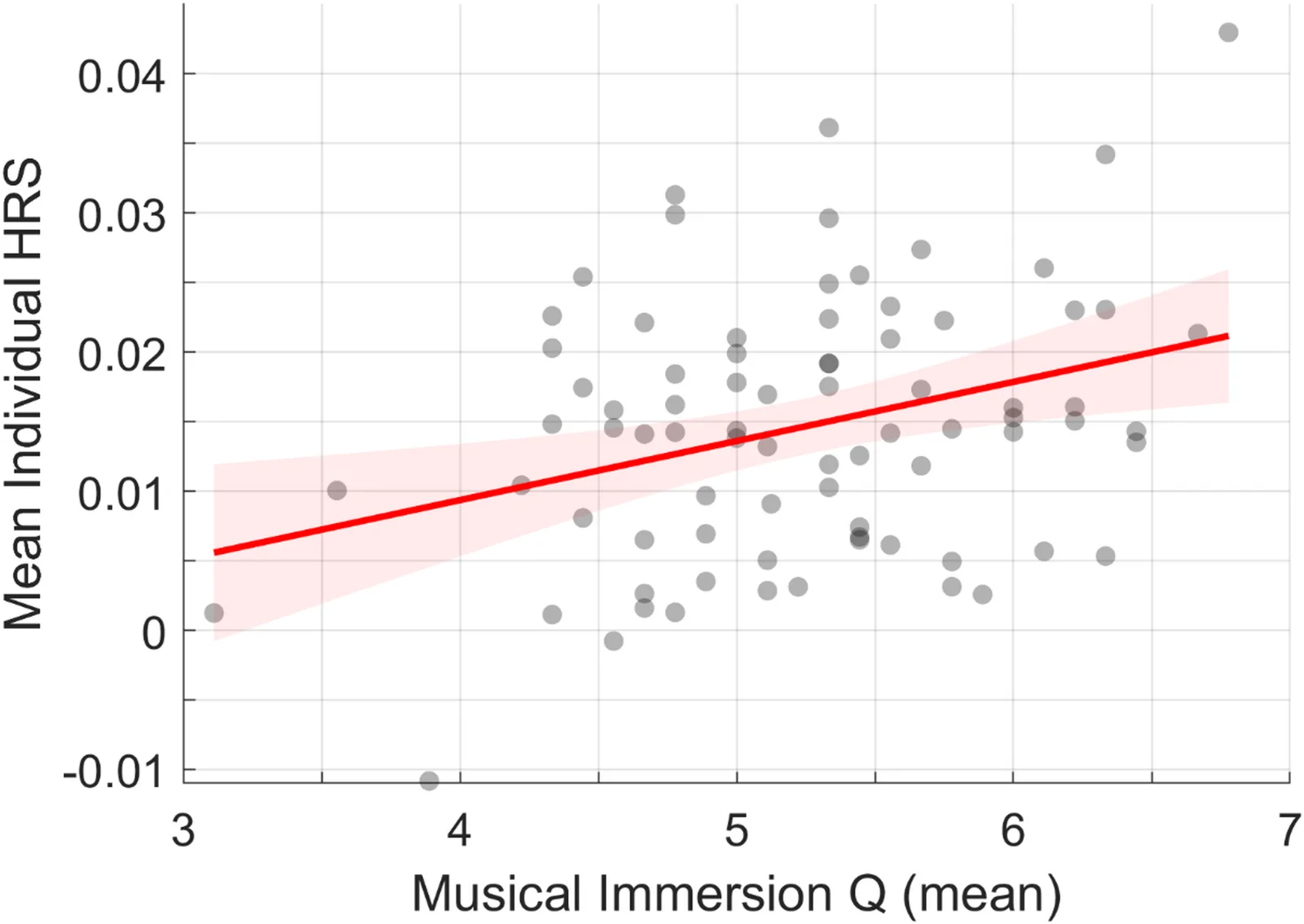
Scatterplot showing significant positive relationship between heart-rate synchrony and self-reported musical immersion.

Following this procedure, only the mean score of the musical engagement questionnaire (see Methods) emerged as significantly predictive of individual heart-rate synchrony (β = 0.0042, SE = 0.014, t(78) = 2.989, *p* = 0.0037). This model (*F*(1, 78) = 8.93, *p* = 0.0037) explained 10.3% of the variance in heart-rate synchrony (R^2^ = 0.103, adjusted R^2^ = 0.091). This relationship is shown in Fig. 4.

### Seating position

A linear regression showed dyadic heart-rate synchrony was not predicted by Euclidian dyadic seating distance between dyad members; (β < 0.001, t(822) = − 0.867, *p* = 0.386). We additionally examined the effect of row number (i.e., how far from the stage/screen were participants) on outcome variables. Distance from stage/screen did not predict heart-rate synchrony for the subset of participants fitted with heart-rate monitors. In the full dataset, distance was significantly predicted by the *attention to others* (*β* = 0.625, *p* < 0.001) and *daydream* (*β* = − 0.275, *p* = 0.043) items of the Musical Absorption Inventory.

### Effect of camera angle on heart-rate synchrony

Heart-rate data from *Studio* participants were segmented by the camera angle of the livestream. There were 8 camera angles across the two concerts (4 in each). Mean dyadic HRS was obtained for each camera angle by conducting Surrogate Synchrony (SUSY) analysis on each individual segment (i.e. the time between cuts; *n* = 86 segments). To reflect the short length of many segments in this dataset, SUSY parameters differed from those in the analyses described above. Here, segment length = 5 s; max lag = 2 s. Segments < 10 s were removed from analysis; *n* = 6). For each dyad, a single mean heart-rate synchrony per camera angle was obtained by subtracting the mean pseudo-Z correlation from the mean observed Z-correlation. As the *camera angle* variable was confounded with *concert*, the Z-difference variable was normalized within each concert (by subtracting the mean and dividing by the standard deviation). This normalized Z-difference variable was included as the outcome variable in a linear mixed effects model, with camera angle entered as a fixed effect, and dyad a random intercept.

**Fig. 5:**
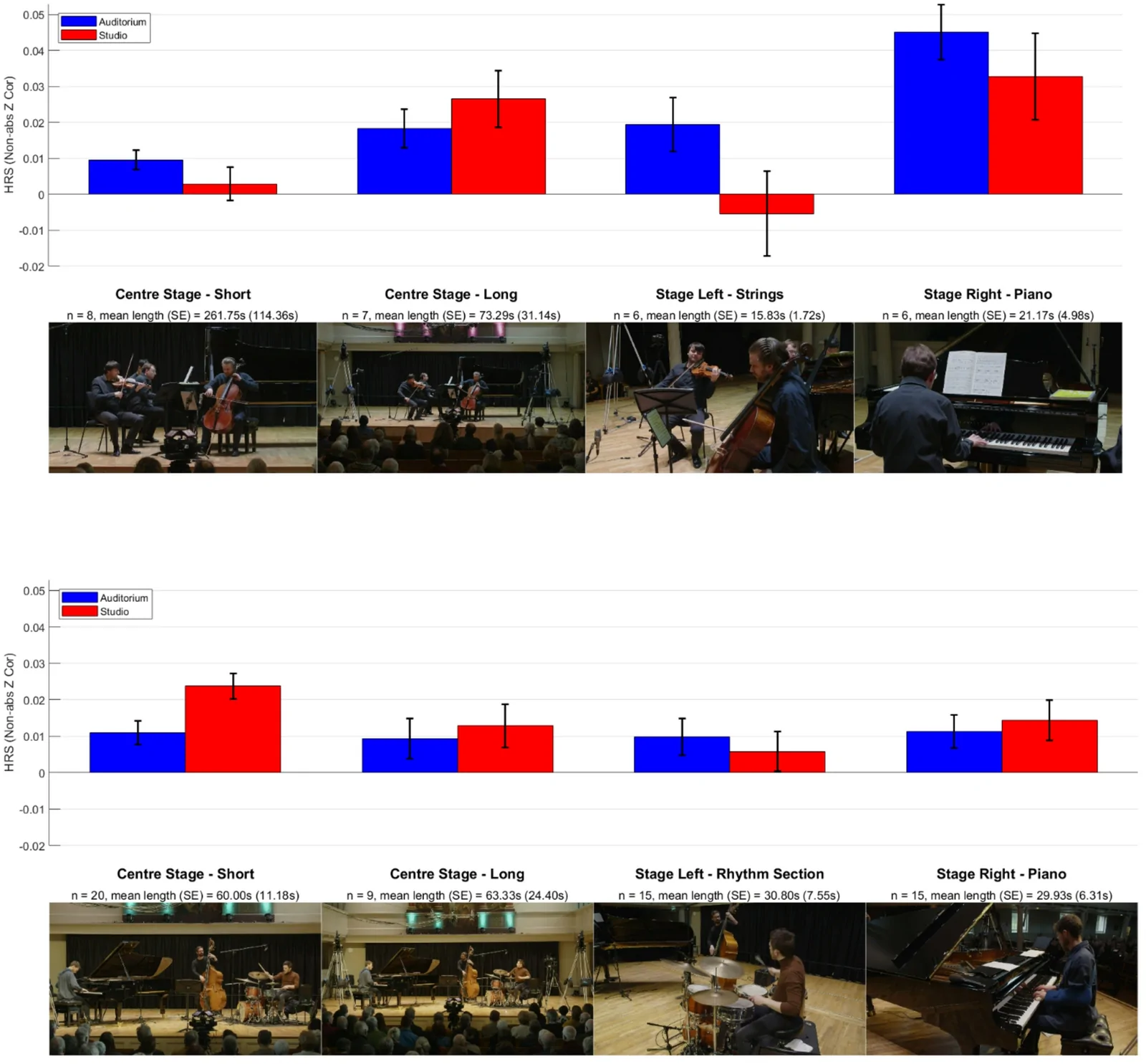
Dyadic heart-rate synchrony (HRS) by camera cut for participants in the livestream (studio) condition (HRS values for the auditorium condition for equivalent segments shown for comparison). Photographs of performers used with consent.

ANOVA applied to the model showed a significant effect of camera angle on dyadic heart-rate synchrony; *F*(7, 1172) = 2.923, *p* = 0.005 (Fig. 5).

Pairwise comparisons using paired t-tests with false-discovery rate correction were conducted to examine which camera angles elicited significantly different HRS levels among the *Studio* audience.

For the classical concert, *Centre Stage - Short* (M = 0.003, SE = 0.005) elicited significantly lower synchrony than *Centre Stage - Long* (M = 0.027, SE = 0.008; t(104) = − 2.49, *p* = 0.036) and *Stage Right - Piano* (M = 0.033, SE = 0.012; t(104) = − 2.46, *p* = 0.036).

*Stage Left - Strings* (M = − 0.005, SE = 0.012) elicited significantly lower synchrony than *Centre Stage - Long* (t(104) = 2.41, *p* = 0.036) and *Stage Right - Piano* (t(104) = − 2.27, *p* = 0.038).

For the jazz concert, *Centre Stage - Short* (M = 0.024, SE = 0.003) elicited significantly higher synchrony than *Stage Left - Rhythm Section* (M = 0.006, SE = 0.005; t(189) = 2.87, *p* = 0.028).

### Sentiment analysis

**Fig. 6:**
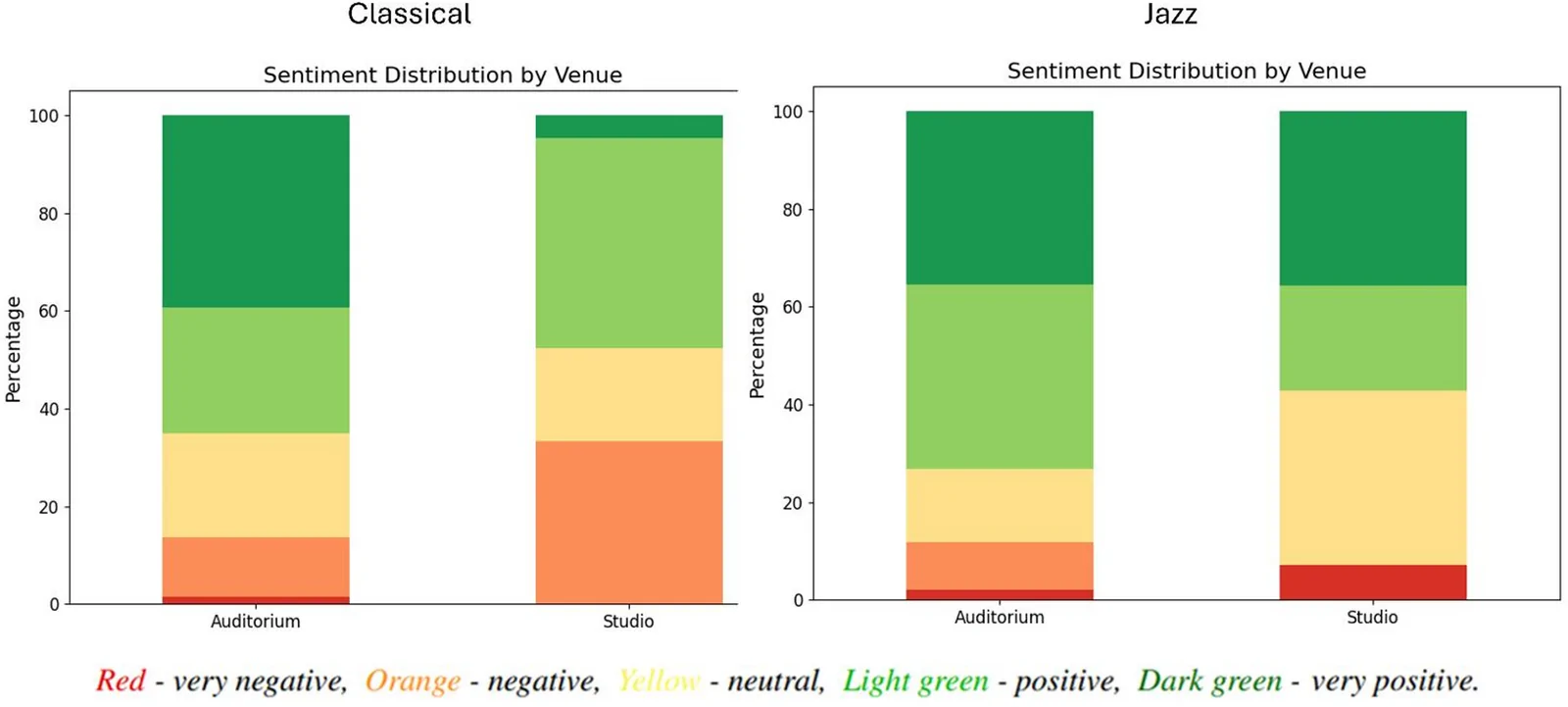
Summary of sentiment analysis.

To statistically assess whether the observed differences in sentiment between venues were meaningful, a Mann–Whitney U test was conducted for each concert day. This non-parametric test was selected due to the ordinal nature of the sentiment data and the independence of the venue groups. For the classical trio, the results revealed a statistically significant difference in sentiment between the Auditorium and the Studio, U = 950.5, *p* = 0.0082. This suggests that the higher sentiment scores in the Main Hall are unlikely to be due to chance. On the other hand, for the jazz trio performance, the difference was not statistically significant, U = 701.0, *p* = 0.6306, indicating that any apparent variation between venues could be attributed to random variation. Output of the sentiment analysis is summarized visually in Fig. 6.

## Discussion

This study compared the audience experience of physically attending a concert compared to watching a livestream version of the same concert. Crucially, as the livestream was viewed from within the same building as the concert, the experience for both audience groups (who were representative of a real paying audience, and randomly assigned to condition) was matched right up to the point of entry to either the main auditorium or adjacent studio. The results indicated that the experience of both groups were significantly different, with the physically-present audience showing significantly increased markers of engagement, both physiological and self-reported, than the livestream audience. These results suggest that there is indeed something ‘special’ about the live experience. However, in this section we will discuss factors that may be cause for due tentativeness in interpreting the results reported.

While the experience of the audience groups, and the audience groups themselves, were as closely matched as possible, we cannot rule out the possibility that the effects are driven by low-level stimulus features or perceptual differences. While the livestream was produced and presented with high-quality professional equipment, the limitation of the audio stereo presentation means that audio quality would have necessarily differed between conditions. While this possibility should be investigated by future studies, it is worth noting that root-mean square energy of classical concerts does not appear to affect HRS^22^, and thus the observed difference in HRS between conditions in the current study is unlikely to have been caused by any difference in audio quality. However, we found that HRS for the livestream audience varied significantly as a function of the choice of camera angle, suggesting that differences in the *visual* experiences of the two conditions may indeed have partly driven results.

The results may also have been affected by the choice of method for synchrony analysis. It has been suggested that group-scale synchrony measures may detect emergent patterns which are obscured in dyad-level analyses, especially for music performance^36^. However, in the current study it was important that individuals’ self-report measures (as well as other individual factors such as seating position) could be examined in relation to physiological synchrony, which necessitated dyad-level synchrony analysis to obtain individual synchrony measures.

We found significant relationships between HRS and many self-report measures (e.g., musical engagement and absorption questionnaires). Individual differences in musicality seemed to have a minimal impact on experience of the concerts. Furthermore, dyadic synchrony was not affected by physical dyadic distance; a finding somewhat at odds with previous findings that interbrain synchrony increases with dyadic proximity^37^.

Interestingly, while seating position did not appear to affect audience experience for the live condition, camera angle did significantly affect the livestream audience’s response. It is worth emphasising the asymmetry this finding reveals: while seating position in the live auditorium had negligible effects on synchrony or reported engagement, camera angle in the livestream condition significantly shaped physiological responses. This suggests that the live experience may confer a form of ‘perceptual robustness’ that is not yet matched by even high-quality mediated alternatives.

In terms of practical applications of findings, and implications for practitioners, and members of the performing arts industry, these results may be encouraging, as they suggest that audiences do in fact have a significantly enhanced experience when viewing live entertainment compared to a livestreamed version. However, potential benefits of viewing livestreamed concerts, such as reduced cost and the comfort and ease of watching from home, were not accounted for in the current study’s design and may outweigh the benefit of the improved experience we found in our study. On the other hand, social benefits associated with the shared experience of live music seem to be a particularly strong motivator for attending live music events (e.g. see^38^; for a review), and in our study the conditions were closely matched in terms of the social experience (e.g., groups that arrived together were seated together regardless of their condition assignment). Thus, it is possible our design is conservative in its estimate in the difference between live and livestreamed experiences.

These results suggest livestream production techniques can be optimized to result in a more positive experience. Choice of camera angle significantly affected HRS, and it is worth noting that the jazz concert, which features much more frequent cuts than the classical concert, seemed to translate better to the livestreamed experience potentially due to the increased number of cuts. While this is a tentative explanation, it is consistent with results from the posthoc analysis; for the jazz concert, the main shot elicited the highest synchrony among all shots for that concert, whereas for the classical concert, the main shot elicited significantly *lower* synchrony than 2 of the other 3 shots, and it seems plausible that the mean duration of these main shots (60 s vs. 262 s) may account for these qualitatively distinct results. These findings suggest that an angle which includes all performers within the shot can be particularly effective, provided it does not linger too long, as it may then potentially have an adverse effect on engagement.

The outcome of our camera angle analysis is consistent with a previous study which found that changes in direction of livestreamed theatrical performances (including cut frequency, angle etc.) can significantly impact audience HRS^9^. One the one hand, live entertainment venues, which in general have struggled to maintain pre-pandemic audience attendance levels (particularly smaller venues^3^) can point to results such as these as evidence that live entertainment is worth the increased cost and effort. On the other hand, compared to home entertainment, these results also suggest that should venues choose to expand their livestreaming capabilities, then it is important that filming choices, such as camera angles and cut frequency, are chosen to optimise viewer experience. The importance of visuals to musical experiences (e.g., music videos) has been shown in previous studies^39^ and this study adds to that literature.

These findings also speak to emerging debates about artificial intelligence (AI)-generated and hybrid performance formats. While an extended discussion of generative AI is outside the scope of this paper, we note that the live advantage we observe (in immersion, reported engagement, and physiological synchrony) suggests something irreplaceable about human-led live performance in an increasingly automated cultural landscape. Similarly, hybrid formats such as holographic concerts (e.g., ABBA Voyage) occupy a middle ground between live and mediated; our results imply that replicating the perceptual affordances of physical co-presence (e.g., spatial presence, peripheral awareness, shared social space) may be critical for such formats to approach live levels of engagement.

In summary, the current study finds evidence that being physically present in a concert audience is associated with a significantly enhanced audience experience compared to viewing a livestream broadcast, even if the livestream is viewed simultaneously to the concert (i.e., it is truly ‘live’) and from within the same venue. However, the mechanisms by which this occurs requires further investigation. It does not seem, based on the results reported, that seating position necessarily makes much of a difference to the live experience, whereas for the livestream experience viewing angle (e.g., as altered by camera angle) *does* significantly impact the experience. This asymmetry - that physical presence buffers against variation in visual perspective, whereas livestream engagement appears critically dependent on editorial framing - represents, to our knowledge, a novel empirical contribution to digital performance theory. It suggests that the ‘liveness’ of an experience may confer a form of perceptual resilience that is not easily replicated via mediated delivery. Future research should examine which perceptual qualities (e.g., spatial audio, peripheral vision, perceptual affordances, haptic cues) underlie this resilience, and how they might be better simulated in livestream production to optimise remote audience experience.

## Supplementary Information

Below is the link to the electronic supplementary material.

Supplementary Material 1

## Data Availability

All data supporting the findings of this study are available on the Open Science Framework (https://osf.io/9cj8e).
