# Supplementary material for: Live music enhances self-reported audience immersion and physiological synchrony compared to live-streaming
Source: Sci Rep. 2026 Jun 30;16:30166. doi: 10.1038/s41598-026-59372-3 (PMC13619450; doi:10.1038/s41598-026-59372-3)
Supplement: Supplementary file 1 [file 41598_2026_59372_MOESM1_ESM.docx]

**Appendix A: Questionnaire (Full Copy)**

**How old are you?**

| 18-24 years | 25-49 years | 50-64 years | 65+ years |
| --- | --- | --- | --- |
| 🞏 | 🞏 | 🞏 | 🞏 |

**Which of the following best describes your gender?**

| Man | Non-binary | Woman | Prefer not to say | Prefer to self-describe |
| --- | --- | --- | --- | --- |
| 🞏 | 🞏 | 🞏 | 🞏 | [……………………………..……....] |

**I engaged in regular, daily practice of musical instrument (including voice) for…**

| 0 years | 1 year | 2 years | 3 years | 4-5 years | 6-9 years | 10+ years |
| --- | --- | --- | --- | --- | --- | --- |
| 🞏 | 🞏 | 🞏 | 🞏 | 🞏 | 🞏 | 🞏 |

**At the peak of my interest, I practiced (hours per day) on my primary musical instrument**

| 0 hours | 0.5 hours | 1 hour | 1.5 hours | 2 hours | 3-4 hours | 5+ hours |
| --- | --- | --- | --- | --- | --- | --- |
| 🞏 | 🞏 | 🞏 | 🞏 | 🞏 | 🞏 | 🞏 |

**In the past 12 months I have attended (number of live music events) as an audience member**

| 0 events | 1 event | 2 events | 3 events | 4-6 events | 7-10 events | 11 or more events |
| --- | --- | --- | --- | --- | --- | --- |
| 🞏 | 🞏 | 🞏 | 🞏 | 🞏 | 🞏 | 🞏 |

**I have had formal training in music theory for (number of years)**

| 0 years | 0.5 year | 1 years | 2 years | 3-5 years | 6-9 years | 10+ years |
| --- | --- | --- | --- | --- | --- | --- |
| 🞏 | 🞏 | 🞏 | 🞏 | 🞏 | 🞏 | 🞏 |

**I can play (number of musical instruments)**

| 0 instruments | 1  instrument | 2 instruments | 3 instruments | 4 instruments | 5 instruments | 6 or more instruments |
| --- | --- | --- | --- | --- | --- | --- |
| 🞏 | 🞏 | 🞏 | 🞏 | 🞏 | 🞏 | 🞏 |

**I listen attentively to music for (amount of time) per day**

| 0-15  minutes | 15-30 minutes | 30-60 minutes | 60-90 minutes | 2  hours | 2-3  hours | 4 hours or more |
| --- | --- | --- | --- | --- | --- | --- |
| 🞏 | 🞏 | 🞏 | 🞏 | 🞏 | 🞏 | 🞏 |

**To what extent did you have these experiences in the concert?**

|  | Not at all | Very little | Little | Some  what | Much | Very much | A lot |
| --- | --- | --- | --- | --- | --- | --- | --- |
| emotion (touched emotionally, surprised, beautiful, deep connection to the music) | 🞏 | 🞏 | 🞏 | 🞏 | 🞏 | 🞏 | 🞏 |
| cognitive appraisal (made me think, expand my understanding of the music) | 🞏 | 🞏 | 🞏 | 🞏 | 🞏 | 🞏 | 🞏 |
| social involvement (feel connected to others, part of the audience) | 🞏 | 🞏 | 🞏 | 🞏 | 🞏 | 🞏 | 🞏 |
| disruptive experiences (disturbed by background noises) | 🞏 | 🞏 | 🞏 | 🞏 | 🞏 | 🞏 | 🞏 |

**During the performance to what extent…**

|  | Not at all | Very little | Little | Some  what | Much | Very much | A lot |
| --- | --- | --- | --- | --- | --- | --- | --- |
| were you absorbed in your own world? | 🞏 | 🞏 | 🞏 | 🞏 | 🞏 | 🞏 | 🞏 |
| Were you absorbed by the music? | 🞏 | 🞏 | 🞏 | 🞏 | 🞏 | 🞏 | 🞏 |
| did you daydream? | 🞏 | 🞏 | 🞏 | 🞏 | 🞏 | 🞏 | 🞏 |
| did you lose your sense of time? | 🞏 | 🞏 | 🞏 | 🞏 | 🞏 | 🞏 | 🞏 |
| were you distracted by thoughts or worries of a personal nature? | 🞏 | 🞏 | 🞏 | 🞏 | 🞏 | 🞏 | 🞏 |
| were you attentive to the music without distracting thoughts, memories, or fantasies? | 🞏 | 🞏 | 🞏 | 🞏 | 🞏 | 🞏 | 🞏 |
| did you pay attention to other audience members? | 🞏 | 🞏 | 🞏 | 🞏 | 🞏 | 🞏 | 🞏 |
| did you pay attention to yourself and your physical sensations? | 🞏 | 🞏 | 🞏 | 🞏 | 🞏 | 🞏 | 🞏 |
| do you feel positively transformed by the music? | 🞏 | 🞏 | 🞏 | 🞏 | 🞏 | 🞏 | 🞏 |
| do you feel negatively transformed by the music? | 🞏 | 🞏 | 🞏 | 🞏 | 🞏 | 🞏 | 🞏 |

**While listening to the music in the concert I…**

|  | Strongly disagree | Disagree | Somewhat disagree | Neither agree nor disagree | Somewhat agree | Agree | Strongly Agree |
| --- | --- | --- | --- | --- | --- | --- | --- |
| was immersed in the sound | 🞏 | 🞏 | 🞏 | 🞏 | 🞏 | 🞏 | 🞏 |
| was captivated by the rhythm | 🞏 | 🞏 | 🞏 | 🞏 | 🞏 | 🞏 | 🞏 |
| was captivated by the musicians’ skill | 🞏 | 🞏 | 🞏 | 🞏 | 🞏 | 🞏 | 🞏 |
| tried to understand the formal structure | 🞏 | 🞏 | 🞏 | 🞏 | 🞏 | 🞏 | 🞏 |
| paid attention to specific instruments | 🞏 | 🞏 | 🞏 | 🞏 | 🞏 | 🞏 | 🞏 |
| felt the music got under my skin | 🞏 | 🞏 | 🞏 | 🞏 | 🞏 | 🞏 | 🞏 |
| felt the music physically | 🞏 | 🞏 | 🞏 | 🞏 | 🞏 | 🞏 | 🞏 |
| felt like crying | 🞏 | 🞏 | 🞏 | 🞏 | 🞏 | 🞏 | 🞏 |
| listened with feeling | 🞏 | 🞏 | 🞏 | 🞏 | 🞏 | 🞏 | 🞏 |

**During the performance I was very immersed:**

| Strongly |  | Somewhat | Neither agree | Somewhat |  | Strongly |
| --- | --- | --- | --- | --- | --- | --- |
| disagree | Disagree | disagree | nor disagree | agree | Agree | Agree |
| 🞏 | 🞏 | 🞏 | 🞏 | 🞏 | 🞏 | 🞏 |

**In the past 12 months how many events have you attended at St George’s as an audience member**

| 0 events | 1 event | 2 events | 3 events | 4-6 events | 7-10 events | 11 or more events |
| --- | --- | --- | --- | --- | --- | --- |
| 🞏 | 🞏 | 🞏 | 🞏 | 🞏 | 🞏 | 🞏 |

**What percentage of the music that you heard today have you heard before**

| 0% | 1-20% | 20-30% | 30-40% | 50-60% | 70-80% | 90-100% |
| --- | --- | --- | --- | --- | --- | --- |
| 🞏 | 🞏 | 🞏 | 🞏 | 🞏 | 🞏 | 🞏 |

**How likely is it that you would consider paying to see this group perform again**

| Never | Extremely unlikely | Unlikely | Neutral | Likely | Extremely Likely | Definitely |
| --- | --- | --- | --- | --- | --- | --- |
| 🞏 | 🞏 | 🞏 | 🞏 | 🞏 | 🞏 | 🞏 |

**How likely is it that you would listen to an audio recording of this group**

| Never | Extremely unlikely | Unlikely | Neutral | Likely | Extremely likely | Definitely |
| --- | --- | --- | --- | --- | --- | --- |
| 🞏 | 🞏 | 🞏 | 🞏 | 🞏 | 🞏 | 🞏 |

**Please use this space to tell us anything else about your experience**

**Thank you for completing this questionnaire**

**Appendix B: List of predictors used in stepwise regression**

**Musicality**

- Average of: "I engaged in regular, daily practice of musical instrument (including voice) for…", "At the peak of my interest, I practiced (hours per day) on my primary musical instrument", "I have had formal training in music theory for (number of years)", "I can play (number of musical instruments)", and "I listen attentively to music for (amount of time) per day"

**Aesthetic Experience - Emotion**

- "To what extent did you have these experiences in the concert? - emotion (touched emotionally, surprised, beautiful, deep connection to the music)"

**Aesthetic Experience - Cognitive**

- "To what extent did you have these experiences in the concert? - cognitive appraisal (made me think, expand my understanding of the music)"

**Aesthetic Experience - Social**

- "To what extent did you have these experiences in the concert? - social involvement (feel connected to others, part of the audience)"

**Aesthetic Experience - Disruption**

- "To what extent did you have these experiences in the concert? - disruptive experiences (disturbed by background noises)"

**Absorption**

- Average of: "During the performance to what extent… Were you absorbed by the music?", "did you lose your sense of time?", and "were you attentive to the music without distracting thoughts, memories, or fantasies?"

**Musical Engagement Questionnaire**

- Mean score of all items in the "While listening to the music in the concert I…" section

**Immersion**

- "During the performance I was very immersed"

**Familiarity with Music**

- "What percentage of the music that you heard today have you heard before"

**Intent - Listen:**

- "How likely is it that you would listen to an audio recording of this group"

**Intent – Pay to See:**

- "How likely is it that you would consider paying to see this group perform again"

**Own World**

- "During the performance to what extent… were you absorbed in your own world?"

**Daydream**

- "During the performance to what extent… did you daydream?"

**Distracted**

- "During the performance to what extent… were you distracted by thoughts or worries of a personal nature?"

**Attention to Others**

- "During the performance to what extent… did you pay attention to other audience members?"

**Attention to Sensations**

- "During the performance to what extent… did you pay attention to yourself and your physical sensations?"

**Positive Transformation**

- "During the performance to what extent… do you feel positively transformed by the music?"

**Negative Transformation**

- "During the performance to what extent… do you feel negatively transformed by the music?"

**Venue Audience Membership**

- "In the past 12 months how many events have you attended at St George’s as an audience member"
